# A Hardware-Friendlyand High-Efficiency H.265/HEVC Encoder for Visual Sensor Networks

**DOI:** 10.3390/s23052625

**Published:** 2023-02-27

**Authors:** Chi-Ting Ni, Ying-Chia Huang, Pei-Yin Chen

**Affiliations:** Department of Computer Science and Information Engineering, National Cheng Kung University, Tainan 70101, Taiwan

**Keywords:** visual sensor networks, HEVC, fast CU partition, texture based, hardware friendly

## Abstract

Visual sensor networks (VSNs) have numerous applications in fields such as wildlife observation, object recognition, and smart homes. However, visual sensors generate vastly more data than scalar sensors. Storing and transmitting these data is challenging. High-efficiency video coding (HEVC/H.265) is a widely used video compression standard. Compare to H.264/AVC, HEVC reduces approximately 50% of the bit rate at the same video quality, which can compress the visual data with a high compression ratio but results in high computational complexity. In this study, we propose a hardware-friendly and high-efficiency H.265/HEVC accelerating algorithm to overcome this complexity for visual sensor networks. The proposed method leverages texture direction and complexity to skip redundant processing in CU partition and accelerate intra prediction for intra-frame encoding. Experimental results revealed that the proposed method could reduce encoding time by 45.33% and increase the Bjontegaard delta bit rate (BDBR) by only 1.07% as compared to HM16.22 under all-intra configuration. Moreover, the proposed method reduced the encoding time for six visual sensor video sequences by 53.72%. These results confirm that the proposed method achieves high efficiency and a favorable balance between the BDBR and encoding time reduction.

## 1. Introduction

Rapid technological developments have increased the demand for sensor networks, including multimedia data sensors. Sensor networks in camera equipment are known as visual sensor networks (VSNs). VSN nodes can capture and send visual data for monitoring applications such as security surveillance, wildlife observation, and object recognition [1,2,3]. Although visual data can enrich monitoring, visual sensors generate vastly more data than scalar sensors. Storing, transmitting, and processing these visual data is challenging due to storage, computing power, and transmission bandwidth limitations [4,5,6]. Therefore, achieving a high compression rate and low complexity are both key requirements of VSNs [7,8,9].

High-efficiency video coding (HEVC/H.265) [10] was developed by the Joint Collaborative Team on Video Coding (JCT-VC), an joint effort by the ITU-T Video Coding Experts Group (VCEG) and the ISO/IEC Moving Picture Experts Group (MPEG). The newest video compression standard, versatile video coding (VVC/H.266) [11], was finalized on July 2020, and the compression complexity of H.266 is substantially greater than that of HEVC. Compared with H.264/AVC [12], HEVC can achieve the same video quality at approximately 50% of the bit rate; this compression of visual data is highly efficient. Hence, the storage, computing power, and transmission bandwidth limitations suggest that H.265 is appropriate for VSNs. Additionally, considering that packet loss may occur during the transmission, these error data may cause error propagation due to inter prediction and motion compensation. Intra-frame encoding plays a vital role in the prevention of the error propagation, because it does not need to reference other previous coded frames [13]. Moreover, due to the large number of computations associated with motion estimation in inter prediction, the HEVC inter coding profile may not be adopted in video applications with a low complexity requirement [14]. HEVC includes many tools to improve the compression efficiency of intra frames, such as coding tree units (CTUs), intra prediction, and rate distortion optimization (RDO). Although these technologies can achieve this high compression rate, they also increase the complexity of compression. Hence, applying HEVC in VSNs requires improvements in encoding efficiency.

To solve this problem, we first analyzed the complexity of intra prediction with various coding unit (CU) sizes by using standard test sequences. Then, we analyzed various video characteristics to design the proposed algorithm. The videos captured by the visual sensor camera usually have a single scene and a restricted directional sensing field of view. The content of the videos usually contain a huge number of homogenous regions, such as the background. In intra prediction, these homogenous regions tend to be larger-sized CUs. On the basis of this analysis, we propose a low-complexity and hardware-friendly accelerating algorithm for HEVC intra encoding that reduces the computational complexity. The key contributions of this work are summarized as follows:

- A hardware-friendly and high-efficiency H.265/HEVC encoder for intra frames is proposed. The proposed method also can be parallelized because it only uses information from the current CU. The proposed method significantly reduces computation complexity while achieving a high compression rate, satisfying the requirements for VSN video transmission.
- Four projection directions are used in the proposed method to predict the depth range of the current CTU and eliminate impossible intra prediction modes. Moreover, to reduce the effects of noise, we normalized the average intensity of each CU to generate a generalized threshold.
- The proposed method achieves high-efficiency encoding; it has more consistent encoding time savings for all test sequences and a slight increase in the Bjontegaard delta bit rate (BDBR) compared to the HEVC test model.

In this study, we provide a hardware-friendly and high-efficiency HEVC encoder to reduce computational complexity for VSN applications. The remainder of the article is organized as follows. Section 2 describes some well-known HEVC acceleration methods. Subsequently, we describe a preliminary analysis of the complexity of the intra prediction and CU partitioning with various CU sizes by using standard test sequences. A hardware-friendly and high-efficiency method developed on the basis of this analysis is described in Section 3. Section 4 reveals that the proposed method achieved high efficiency and a favorable balance between the BDBR [15] and encoding time reduction for the test sequences of the HEVC test model.

## 2. Related Work

Some recent studies have attempted to reduce the computational complexity of HEVC using a variety of methods, such as fast decision algorithms for CU size and mode prediction methods. Several studies have presented texture feature- or machine learning (ML)-based techniques to reduce redundancy in HEVC encoding. Works exploiting texture features include [16,17,18,19]. In  [16], Min et al. used global and local texture complexity and four local edge complexity metrics for each block to determine partitioning. The information of neighboring CUs was considered in [17,18,19,20]. Shen et al. [17] applied the most probable mode (MPM) method, which compares the current CU depth with that of the above and the remaining CUs and exploits texture complexity to reduce redundant processes. Le et al. [18] used four spatially neighboring CUs that had been encoded to predict the optimal depth. In [19], Lu et al. used the average depth instead of the maximum depth of neighboring CUs to predict a depth range for the current CU. Fengwei et al. [16] proposed an early termination algorithm for CU partition based on statistical analysis and a fast mode selection algorithm based on the best mode distribution characteristics.

In addition to these texture-based approaches, ML methods have also been proposed. Refs. [21,22,23,24,25] used a support vector machine (SVM) to reduce the encoding complexity. Liu et al. [21] used the features of texture complexity, direction complexity, sub-CU information, and the quantization parameter (QP) to determine the CU depth. Zhang et al. [22] used a two-stage SVM method. In the first stage of classification, a three-output classifier with offline learning was developed to enable early termination of deciding the size or checking depth of the current CU. The second stage of binary classification, which performed online learning on previously encoded frames, was proposed to further refine the determination of CU size. Werda et al. [23] designed a fast CU partition module based on the SVM approach and a gradient-based fast intra prediction mode module. In [24], SVM is used for decision making over the selected intra prediction mode classification which significantly reduces the number of modes. Amna et al. [25] built an online SVM-based method to forecast the CU partition module. The convolutional neural network (CNN) has also been applied to accelerate intra mode decision-making. Yi et al. [26] used a CNN to make the intra mode decision by the features of CUs. In [27], CNN was used to predict the depth of CTU. CNN obtains 64 × 64 CTU as input, and the prediction of depth for each 64 × 64 coding unit is represented by a 16 × 16 matrix of each 4 × 4 block. According the depth matrix, the redundancy partitioning is skipped.

Several studies have attempted to accelerate HEVC encoders for VSNs [28] or vehicular ad hoc networks [29]. In [28], Pan et al., analyzed the content properties of CUs to reduce the encoding complexity of an HEVC encoder for VSNs. In [29], an initial coding tree unit depth decision algorithm was developed that controlled the depth search range. Second, a Bayesian classifier was used to predict unit decisions for inter prediction, and the prior probability value was calculated using the Gibbs random field model.

Although these methods can accelerate CU partitioning, the correlation between CU and texture features has rarely been exploited, and some proposed algorithms are not suitable for hardware implementation. Therefore, methods that strike a better trade-off between complexity reduction and encoding loss can still be formulated. In the next section, we formally examine intra prediction and CU partitioning complexity and then present a hardware-friendly and high-efficiency method.

## 3. Proposed Method

In this section, the original HEVC encoding process is introduced, and the proposed accelerating algorithm for reducing the computation complexity of this process is then described.

### 3.1. Encoding Process in HEVC

**Figure 1:**
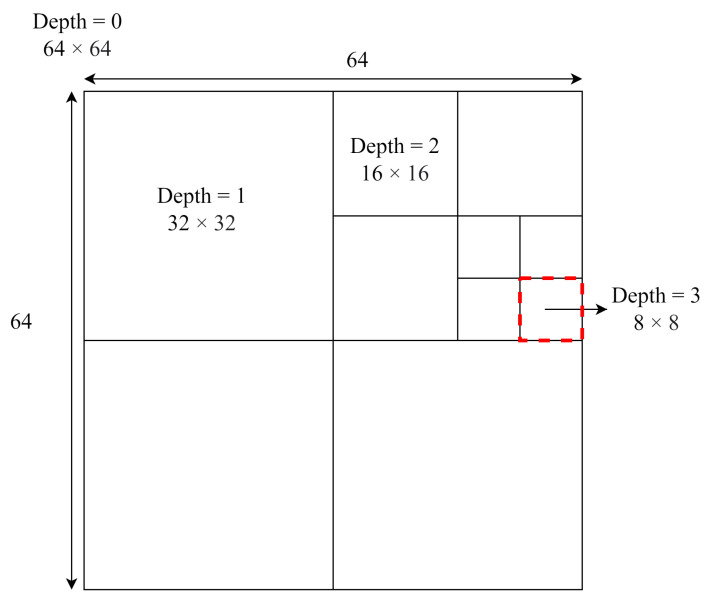
Example of CTU partition.

**Figure 2:**
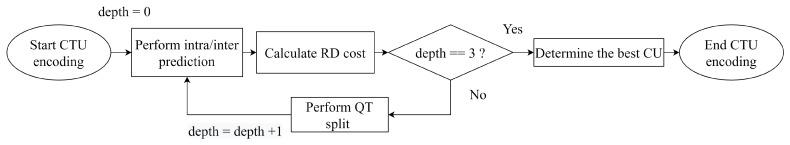
Flowchart of HEVC CTU encoding.

HEVC is based on a block-based hybrid coding architecture. Each frame of an input video is divided into numerous blocks, called CTUs, and each CTU is divided into many smaller blocks called CUs. The size of a CTU is 64 × 64 and it can be split using a quadtree; this partitioning is displayed in Figure 1. CUs can be classified as having one of four depths: 64 × 64, 32 × 32, 16 × 16, or 8 × 8. A total of 85 CUs can be examined during CTU encoding. As presented in Figure 2, for each CU, intra and inter prediction must be performed before rate–distortion optimization (RDO) [30] is executed to calculate the rate–distortion cost (RD cost). Finally, the encoding scheme with the minimal RD cost is selected as the optimal encoding method. The RD cost is expressed as (1), where $RD_{cost}$ is the RD cost, λ is the Lagrange multiplier, *R* is the number of encoding bits, and *D* is the reconstruction distortion.

(1) $$RD_{cost}=D+\lambda \times R$$

To select the optimal encoding scheme, all possible depth levels and prediction modes must be exhaustively checked. The recursive structure of CUs results in many redundant computational steps; this restricts the scheme’s ability to be used in HEVC applications.

### 3.2. A Hardware-Friendly and High-Efficiency H.265/HEVC Encoder for Visual Sensor Networks

To accelerate these recursive computations, we propose an algorithm for CU partitioning and intra prediction. The proposed algorithm is based on the main idea of intra prediction, which can encode and decode individually without referencing the information from other frames. Additionally, to reference information from the previous frame, extra designs must be added to the hardware architecture. Considering the hardware cost, referencing information from only the current frame is more hardware friendly. Hence, the proposed algorithm in our design does not use the information from the previous frame. The proposed method has three steps: edge feature extraction, projected gradient normalization, and finally, fast CU partition and mode decision. These steps are detailed in the following sections.

#### 3.2.1. Edge Feature Extraction

Due to the limitations of the VSN and hardware implementation, we adopted filters for extracting features instead of applying machine learning. We adopted the edge detection operator and calculated the gradient of four directions ($0^{\circ }$, $45^{\circ }$, $90^{\circ }$, $135^{\circ }$) by projecting G(i,j). These four gradients were denoted as $D_{0},D_{45},D_{90}$, and $D_{135}$. $G_{0}\left(i,j\right)$, $G_{90}\left(i,j\right)$ and G(i,j) were calculated by Equations (2)–(4), where (2) and (3) are the 3 × 3 intensity matrices of the current block *A* that are centered on the point currently being computed. G(i,j) is the gradient value as calculated from $G_{0}\left(i,j\right)$ and $G_{90}\left(i,j\right)$. *i* and *j* represent the position of the current center pixel in a row and in a column, respectively.

In general, $G_{0}\left(i,j\right)$, $G_{90}\left(i,j\right)$ can be used to calculate the four directions by applying θ(i,j) as expressed in Equation (5). The gradient of each direction by the projected G(i,j) and θ(i,j) can be calculated by Equations (6)–(9).

(2) $$G_{0}\left(i,j\right)=A_{i,j}\times \left[\begin{array}{ccc} 1 & 2 & 1\\ 0 & 0 & 0\\ -1 & -2 & -1 \end{array}\right]$$

(3) $$G_{90}\left(i,j\right)=A_{i,j}\times \left[\begin{array}{ccc} -1 & 0 & 1\\ -2 & 0 & 2\\ -1 & 0 & 1 \end{array}\right]$$

(4) $$G\left(i,j\right)=\sqrt{G_{0}{\left(i,j\right)}^{2}+G_{90}{\left(i,j\right)}^{2}}$$

(5) $$\theta \left(i,j\right)=\arctan\left(\frac{G_{0}\left(i,j\right)}{G_{90}\left(i,j\right)}\right)$$

(6) $$D_{0}\left(i,j\right)=G\left(i,j\right)\times \sin\theta \left(i,j\right)$$

(7) $$D_{45}\left(i,j\right)=G\left(i,j\right)\times \sin\theta \left(i,j\right)\times \frac{\sqrt{2}}{2}-G\left(i,j\right)\times \cos\theta \left(i,j\right)\times \frac{\sqrt{2}}{2}$$

(8) $$D_{90}\left(i,j\right)=G\left(i,j\right)\times \cos\theta \left(i,j\right)$$

(9) $$D_{135}\left(i,j\right)=G\left(i,j\right)\times \sin\theta \left(i,j\right)\times \frac{\sqrt{2}}{2}+G\left(i,j\right)\times \cos\theta \left(i,j\right)\times \frac{\sqrt{2}}{2}$$

**Figure 3:**
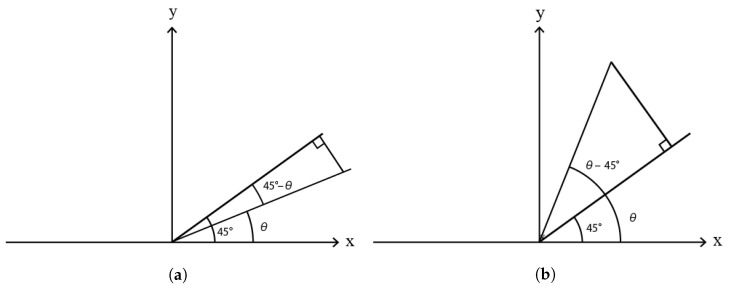
Projected gradient of $45^{\circ }$. (**a**) Projected gradient when θ is less than $45^{\circ }$; (**b**) Projected gradient when θ is bigger than $45^{\circ }$.

**Figure 4:**
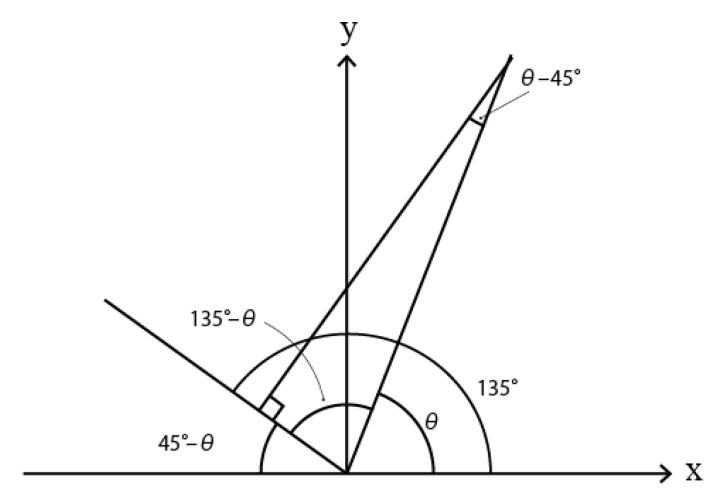
Projected gradient of $135^{\circ }$.

$D_{45}$ can be derived as shown in Figure 3. The projection formula can be expressed as G(i,j)×sin(θ−45) or G(i,j)×sin(45−θ). The absolute value of these expressions are the same; hence, we adopt only one of them to calculate the gradient. $D_{135}$ can also be derived from Figure 4. The projection formula be expressed as G(i,j)×cos(θ−45).

To reduce computational complexity, $D_{0}$ and $D_{90}$ can be reduced using Equations (10), (11), and (4). Hence, Equations (6) and (8) can be rewritten as Equations (12) and (13). Through the application of Equations (12) and (13), Equations (7) and (9) can be rewritten as Equations (14) and (15). These manipulations greatly reduce the computational complexity. The above steps can be summarized in Algorithm 1. The gradient of the four directions ($D_{0},D_{45},D_{90},D_{135}$) by projecting can be calculated using $G_{0}$ and $G_{90}$.

(10) $$\cos\left(\arctan\left(\frac{G_{0}\left(i,j\right)}{G_{90}\left(i,j\right)}\right)\right)=\frac{1}{\sqrt{1+{\left(\frac{G_{0}\left(i,j\right)}{G_{90}\left(i,j\right)}\right)}^{2}}}$$

(11) $$\sin\left(\arctan\left(\frac{G_{0}\left(i,j\right)}{G_{90}\left(i,j\right)}\right)\right)=\frac{{\left(\frac{G_{0}\left(i,j\right)}{G_{90}\left(i,j\right)}\right)}^{2}}{\sqrt{1+{\left(\frac{G_{0}\left(i,j\right)}{G_{90}\left(i,j\right)}\right)}^{2}}}$$

(12) $$D_{0}\left(i,j\right)=G_{0}$$

(13) $$D_{90}\left(i,j\right)=G_{90}$$

(14) $$D_{45}\left(i,j\right)=G_{0}\times \frac{\sqrt{2}}{2}-G_{90}\times \frac{\sqrt{2}}{2}$$

(15) $$D_{135}\left(i,j\right)=G_{0}\times \frac{\sqrt{2}}{2}+G_{90}\times \frac{\sqrt{2}}{2}$$

**Algorithm** **1** Projection of each pixel.**Input:** Original image *A*; **Output:**
$D_{0}\left(i,j\right),D_{45}\left(i,j\right),D_{90}\left(i,j\right),D_{135}\left(i,j\right)$ for each (i,j) in image *A*
1:initial i=0 and j=0;2:**for** each (i,j)∈A **do**3:     Compute $G_{0}\left(i,j\right)$, $G_{90}\left(i,j\right)$ with Equations (2) and (3);4:     Compute $D_{0}\left(i,j\right),D_{45}\left(i,j\right),D_{90}\left(i,j\right),D_{135}\left(i,j\right)$ with Equations (12)–(15);5:**end for**6:**return**$D_{0}\left(i,j\right),D_{45}\left(i,j\right),D_{90}\left(i,j\right),D_{135}\left(i,j\right)$;


For each direction, the projection of each pixel is accumulated in the block. The gradient sum $Gs_{d}$ in each direction is expressed as Equation (16).

(16) $$Gs_{d}=\sum_{i=0}^{H}\sum_{j=0}^{W}\left|(D_{d}\left(i,j\right))\right|,d=0,45,90,135$$

where *W* represents the width of the block, and *H* represents the height of the block. The direction with the greatest sum is the main direction of the block.

#### 3.2.2. Projected Gradient Normalization

**Figure 5:**
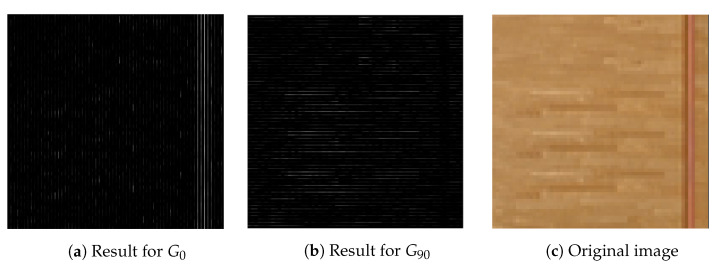
Direction error example.

As mentioned in the previous section, the main direction of the block can be calculated by Equation (16); however, each direction is correlated. For example, a texture with $G_{0}=10$ and $G_{90}=0$ has the projection values of 10, 7.07, 0, and 7.07 for the directions $0^{\circ }$, $45^{\circ }$, $90^{\circ }$, and $135^{\circ }$, respectively. If we consider all directions, the main direction of the block might not be the direction with the greatest sum, as demonstrated by Figure 5 and Table 1. The texture of the block seems to be vertical or horizontal; however, the main direction calculated by Equation (16) is $45^{\circ }$ or $135^{\circ }$.

To solve this problem, only some instead of all directions are considered, instead of all of them. Equation (17) is introduced to analyze the relationship between each direction and calculate the difference between the greatest projection value $MD_{1}$ and the second-greatest projection value $MD_{2}$. To identify the threshold for determining which group an angle belongs to if it is not near any main direction, we observed a statistical analysis of the relation between the projection of each direction with intensity G(i,j)=10. For example, the projections on $0^{\circ }$ and $45^{\circ }$ are almost identical if the angle is approximately between them; determining which group this angle belongs to is challenging. Hence, we only take $MD_{1}$ as the main direction if $Max_{d}>0.12$. Otherwise, both directions are considered. The relevant equations are Equations (18) and (19), where $d_{1}$ and $d_{2}$ represent the directions with the greatest and second-greatest projection values, respectively. Moreover, to reduce the effect of noise, if the intensity of $D_{0}\left(i,j\right)$, $D_{45}\left(i,j\right)$, $D_{90}\left(i,j\right)$, and $D_{135}\left(i,j\right)$ is too small, it is set to 0.

(17) $$Max_{d}=\left(MD_{1}-MD_{2}\right)/MD_{1}$$

(18) $$\begin{array}{c} Dn_{d}\left(i,j\right)=\left\{\begin{array}{cc} D_{d}\left(i,j\right), & d=d_{1},d_{2}\\ 0, & else \end{array}\right. \end{array}$$

(19) $$\begin{array}{c} Dn_{d}\left(i,j\right)=\left\{\begin{array}{cc} D_{d}\left(i,j\right), & d=d_{1}\\ 0, & else \end{array}\right. \end{array}$$

After the projection value is adjusted, Equation (16) is used to calculate the magnitude of each direction and to sort them from largest to smallest; that is, $M_{1},M_{2},M_{3},$ and $M_{4}$. The direction with the greatest magnitude is the main direction of the block.

#### 3.2.3. Fast CU Partition and Mode Decision

Generally, the CTU partition is based on the complexity and distribution of the texture; hence, the texture complexity can be used to determine whether to halt splitting of the current CU. For example, if a block contains more textures, its edge is more obvious and the average gradient is larger. Typically, the gradient G(i,j) is adopted as in Equation (4); however, both the hardware implementation cost and the computational complexity of this method are overly large. We therefore adopted absolute values to approximate the gradient [31], and Equation (4) can be rewritten as Equation (20).

(20) $$G\left(i,j\right)=\left|G_{0}\left(i,j\right)+G_{90}\left(i,j\right)\right|$$

To judge a homogeneous block, the average intensity is used to represent the texture complexity of the CU and is calculated as in Equation (21), where *N* represents the size of the CU.

(21) $$G_{avg}=\frac{\sum_{i=0}^{N}\sum_{j=0}^{N}G\left(i,j\right)}{N^{2}},N=64,32,16$$

To select a general threshold, we normalized the average intensity of each CU with the same size in the same frame from 0 to 255 as in Equation (22), where $G_{max}$ represents the maximum intensity of all CUs with the same size in the same frame.

(22) $$G_{nor}=\frac{G_{avg}}{G_{max}}\times 255$$

**Figure 6:**
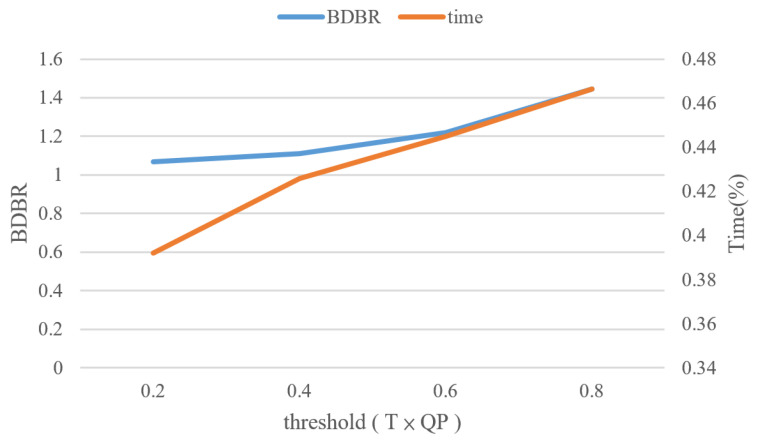
Relationship between BDBR, time reduction, and threshold × QP.

After the normalized gradient is obtained, a threshold is set to determine whether the texture is flat or complex and whether CU splitting should be halted. The quantization parameter (QP) affects the CU partitioning; a smaller QP preserves more detail in the video and tends to cause increased splitting of CUs. Several video sequences were used to study the relationship between the threshold, QP, encoding time reduction, and BDBR. The experimental results are presented in Figure 6; the orange line represents time reduction, and the blue line represents the BDBR. A threshold of 0.4 × QP strikes the best trade-off between BDBR and time reduction. If the $G_{nor}$ of the current CU is smaller than the threshold, partitioning and CU splitting are halted.

**Figure 7:**
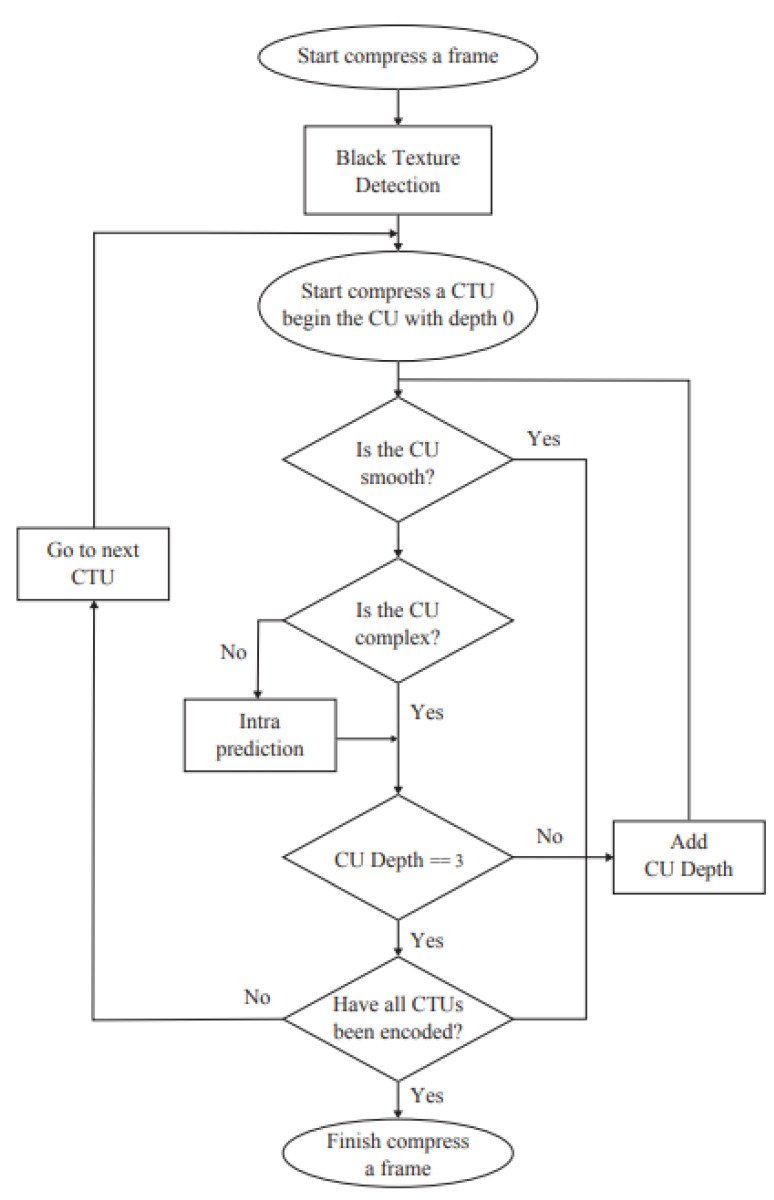
Algorithm for CU partitioning.

If splitting of the current CU is not halted, it must be determined if the intra prediction of the current depth can be skipped for the current CU. In the proposed method, this was performed on the basis of the direction of four sub-CUs. Because the main direction of each CU was calculated, the number of sub-CUs with a direction different from the current CU can be counted. First, sub-CUs are filtered on the basis of the texture intensity; the direction of a sub-CU is considered only if its M1 is greater than 0.25 times M1 of the current CU. We then consider the main direction of the CU and the sub-CU; if these directions differ, the sub-CU is counted. If the count is greater than 2, half of the sub-CUs have a different direction than the current CU, and intra prediction is skipped for the current CU. Figure 7 presents the algorithm for CU partitioning. Subsequently, the intra mode decision-making method is introduced.

The accumulated magnitude of the four CU directions can be obtained from Equation (16) in Section 3.2.2. To determine the main direction of the current CU, Equation (23) is used to calculate the ratio $P_{i}$ of $M_{1}$ and $M_{2}$. If the $M_{2}$ is similar to $M_{1}$ ($P_{i}$ > 0.2), the texture of the current CU is considered to contain two directions. After the main direction of the current CU is calculated, the results on Table 2 and Table 3 are used to obtain the corresponding modes for intra prediction.

(23) $$P_{i}=\frac{M_{i}}{\sum_{j=1}^{4}M_{j}},i=1,2$$

**Figure 8:**
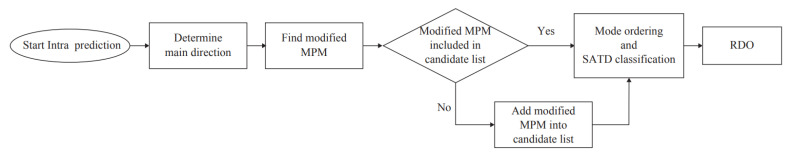
Algorithm for building the mode candidate list.

In addition to these modes, the intra modes of the neighboring CU, direct current (DC) modes, and planar modes are also added to the mode candidate list. Figure 8 presents the algorithm for building the mode candidate list.

**Figure 9:**
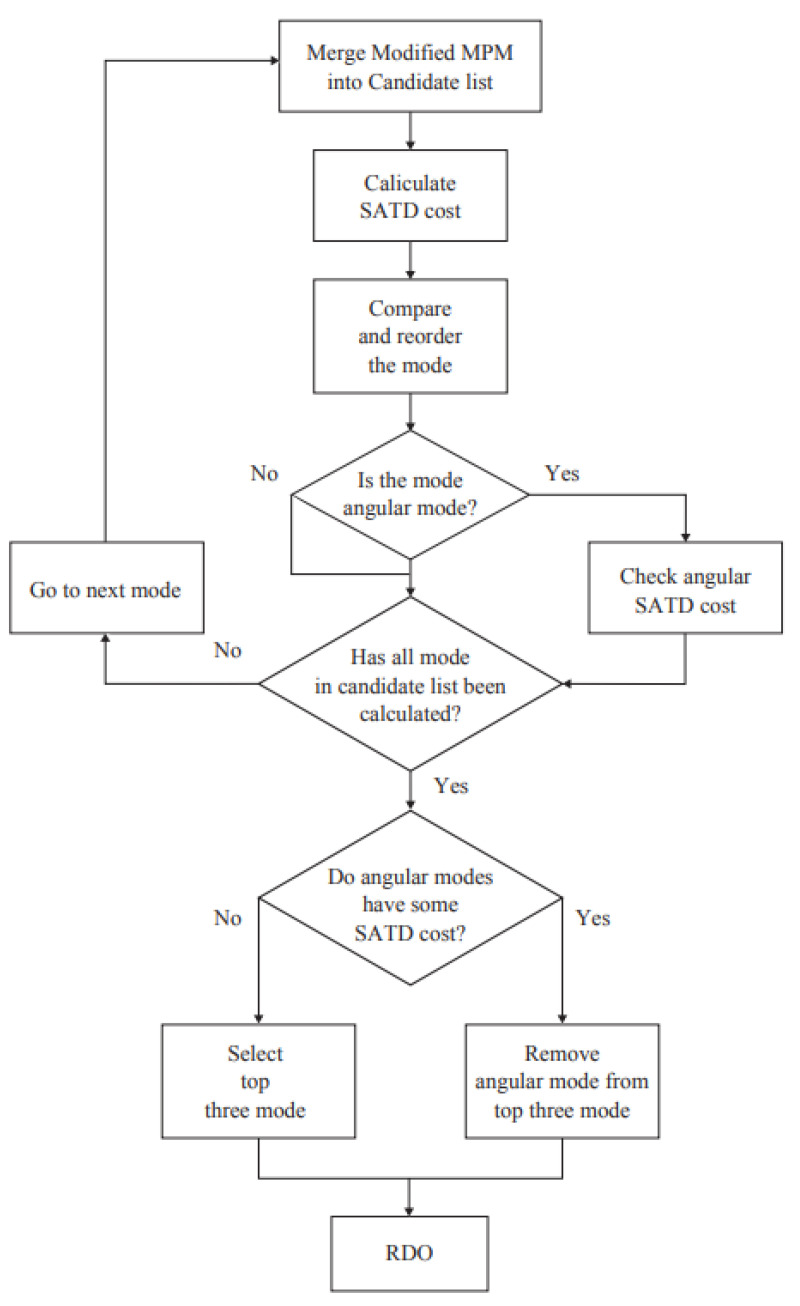
Algorithm for mode ordering and SATD classification.

After obtaining the mode candidate list based on the method of [32], we reordered the modes in the candidate list after calculating the sum of the absolute transform difference (SATD) cost and selected the three modes with the lowest cost as the candidates that undergo the time-consuming RDO process. The SATD cost is calculated by Equation (24), where $D_{SATD}$ is the residual of the SATD, λ is the Lagrange multiplier, and $Bits_{m}$ is the number of bits for the prediction mode. The most suitable mode for the current block will be planar or DC if all angular modes have the same SATD cost. Therefore, all angular modes were removed from the candidate list if all angular modes had the same SATD cost. A flowchart of this process is presented in Figure 9.

(24) $$C_{SATD}=D_{SATD}+\lambda \times Bits_{m}$$

## 4. Experimental Results

We evaluated our proposed method by using HEVC test software (HM) and compared the results with several related works to validate the efficiency of the proposed algorithm. The main reason why we employed the HM encoder is to obtain a fair comparison. To our knowledge, the HM encoder is the recognized standard version, which is employed in most recent studies instead of x265.The proposed algorithm was implemented in HM 16.22 to evaluate its overall performance.

### 4.1. Experimental Environment and Conditions

We used the most recently released version of the HEVC test software to evaluate the algorithms. All tests were performed using the all-intra configuration. The test sequences recommended by JCT-VC [33] from class A to class E were used to evaluate our algorithm in terms of BDBR and time reduction. Time reduction was determined using Equation (25); QPs represents the QP set {22,27,32,37}, $T_{ori}$ is the total encoding time of the HM encoder, and $T_{mod}$ is the total encoding time of the HM encoder with our algorithms. BDBR was determined based on YUV-PSNR and bit rate. The testing machine had an Intel Core i7-8700 CPU clocked at 3.20 GHz and was running Windows 10 (64 bit).

(25) $$TS=\frac{1}{4}\sum_{q\in QPs}\frac{T_{ori}\left(q\right)-T_{mod}\left(q\right)}{T_{ori}\left(q\right)}\times 100$$

### 4.2. Experimental Results

To evaluate the performance of each individual method, several test videos were used. The results are summarized in Table 4 and Table 5. Table 4 presents the results for the proposed method with normalization and without normalization. The normalization is effective when the brightness of video is low or the complexity of content is quite different, such as with Mobisode2, Keiba, and Johnny in Table 4. The proposed method with normalization can obtain a better BDBR with little time savings loss. A video with an average brightness will not be affected by normalization, such as BlowingBubbles in Table 4. Table 5 presents the results for acceleration of CU partition and acceleration of CU partition and intra mode, respectively. The acceleration of CU partition reduced the complexity of the encoding process by approximately 40% on average, with a slight increase in BDBR. The results also reveal that the acceleration of the intra mode can reduce encoding time by approximately 10% and negligibly reduce BDBR.

Table 6 and Table 7 present the results for the proposed method and previous methods [22,24,25,34,35]. The proposed method reduced the encoding time by 45.33% on average and increased the BDBR by 1.07% when compared to HM 16.22. The symbol * indicates that some frames of a sequence were used in the training set in [22]. As indicated in Table 6 and Table 7, the time savings of the algorithms of Zhang et al. [22], Jamali et al. [34], Sulochana et al. [24], Amna et al. [25], and Yin et al. [35] were 48.02%, 47.0%, 31.9%, 47.0%, and 32.6% on average, respectively, and their average BDBR increased by 1.39%, 1.44%, 0.83%, 1.5%, and 0.87%, respectively. The BDBR of the proposed method was lower than those of [22,25,34], indicating that the proposed method ensures that the most CUs are predicted correctly. In addition, to balance the performance of BDBR and TS, we adopted the TS/ BDBR to evaluate the performance better. This evaluation metric is also used in [36,37,38], so we can obtain an intuitive evaluation of the results. Table 8 demonstrates that evaluation measure for the proposed method and previous methods [22,24,25,34,35]. The results reveal that under the same increase in BDBR increase, the time savings of our proposed method are the best.

**Figure 10:**
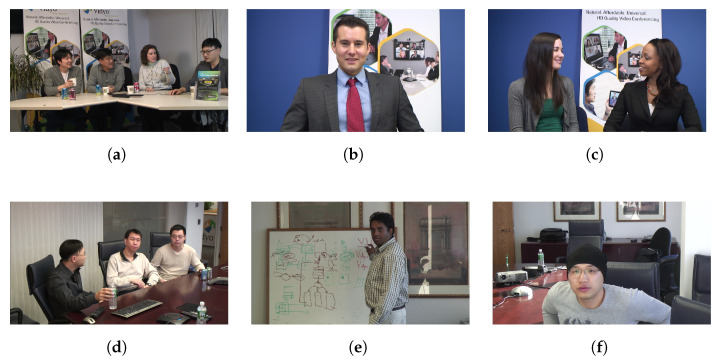
The test sequences. (**a**) FourPeople; (**b**) Johnny; (**c**) KristenAndSara; (**d**) Vidyo1; (**e**) Vidyo3; (**f**) Vidyo4.

**Figure 11:**
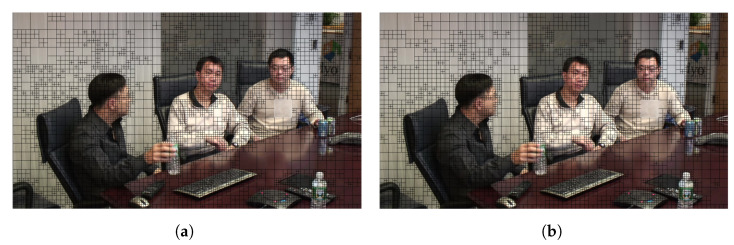
CU partition results of various algorithms. (**a**) Partition of Vidyo1 by using HM16.22. (**b**) Partition of Vidyo1 by using our method.

In addition to these test video sequences, according to the characteristics of VSNs, cameras capture videos of distant objects/scenes from a certain direction [3], and six video sequences are taken from [28] as visual sensor videos to evaluate the proposed algorithm. Hence, the six visual sensor videos, namely FourPeople, Johnny, KristenAndSara, Vidyo1, Vidyo3, and Vidyo4, were used to evaluate the proposed method. The six video sequences are displayed in Figure 10. Each video sequence has a resolution of 1280 × 720 and frame rate of 60 fps. Table 9 presents the results for the proposed method and [22,34]. The proposed method reduces the encoding time by 53.72% and increases BDBR by 1.13% on average compared with HM 16.22. Although the time reduction obtained in Zhang et al. [22] is 8% higher than the proposed method, its BDBR is twice as high. Moreover, the BDBR and time savings of the proposed method are both superior to the algorithm of Jamali et al. [34]. Table 9 demonstrates that the proposed method achieved a higher efficiency and a better balance between BDBR and time reduction for VSNs than previous algorithms. Figure 11 illustrates the splitting results for the default HM16.22 algorithm and the proposed method with QP set to 22. The CU partition is skipped if the block is flat, and the split is close to the textures if the block is complex.

## 5. Conclusions

In this paper, a hardware-friendly and high-efficiency H.265/HEVC encoder for VSN is proposed. The proposed method exploits the gradient of the texture to skip redundant CU partitioning processes and facilitates efficient intra prediction. The experimental results reveal that the proposed method can reduce the encoding time by 45.33% but only increases BDBR by 1.07% when compared to HM16.22. Moreover, the performance of the proposed method for six visual sensor video sequences was superior to that of previous algorithms. In summary, our proposed method achieves high-efficiency encoding with more consistent encoding time reductions for all test sequences and only a small increase in BDBR.

HEVC is a block-based hybrid coding architecture; in addition to intra prediction, there is also inter prediction configuration. Based on the experience gained in the development of the proposed method, an acceleration algorithm for inter prediction or other block-based hybrid coding architectures can be developed.

## Figures and Tables

**Table 1:** Magnitude of the four directions in Figure 5.

Direction	$0^{\circ }$	$45^{\circ }$	$90^{\circ }$	$135^{\circ }$
Magnitude	35,423.4	37,402.9	31,843.2	37,967.3

**Table 2:** Candidate modes for one main direction.

Main Direction	$0^{\circ }$	45${}^{\circ }$	$90^{\circ }$	$135^{\circ }$
Prediction mode	6∼14	30∼34, 2∼6	22∼30	14∼22

**Table 3:** Candidate modes for two main directions.

Main Direction	$0^{\circ }$ and $45^{\circ }$	$45^{\circ }$ and $90^{\circ }$	$90^{\circ }$ and $135^{\circ }$	$0^{\circ }$ and $135^{\circ }$	$0^{\circ }$ and $90^{\circ }$	$45^{\circ }$ and $135^{\circ }$
Prediction mode	2∼10	26∼34, 2	18∼26	10∼18	8∼12, 24∼28	32∼34, 2∼4, 16∼20

**Table 4:** The encoding performance of the proposed normalizing method with HM 16.22.

Test Sequence	With Normalize		Without Normalize	
	BD-Rate (%)	TS (%)	BD-Rate (%)	TS (%)
Flowervase	1.23	38.1	1.51	45.1
BlowingBubbles	1.11	40.7	1.11	41.0
Mobisode2	0.45	62.2	9.37	78.1
Keiba	0.46	61.4	1.62	62.2
Johnny	1.20	53.6	1.81	61.4

**Table 5:** The encoding performance of the proposed individual method with HM 16.22.

Test Sequence	CU Partition		CU Partition + Intra Mode	
	BD-Rate (%)	TS (%)	BD-Rate (%)	TS (%)
BlowingBubbles	0.89	35.6	1.11	40.7
BasketballDrill	0.71	35.7	0.72	41.0
RaceHorses	0.59	36.8	0.70	44.1
Johnny	1.05	41.2	1.20	53.6
FourPeople	1.08	42.1	1.17	51.0
BasketballDrive	0.72	39.1	0.87	51.3
ParkScene	0.84	36.2	0.94	43.0
Traffic	0.82	37.4	0.91	44.6

**Table 6:** The time savings of the proposed method compared with the anchor method and previous works.

Class	Test Sequence	Proposed	[22]	[24]	[25]	[34]	[35]
		TS (%)	TS (%)	TS (%)	TS (%)	TS (%)	TS (%)
2560 × 1600 Class A	Traffic	44.6	45.6	32.2	-	48.8	-
	PeopleOnStreet	44.2	44.8	-	-	49.4	-
1920 × 1080 Class B	BasketballDrive	51.3	61.0	30.2	53.6	49.1	39.6
	BQTerrace	43.4	51.0	-	46.1	46.7	25.4
	Cactus	44.0	45.5	-	45.8	47.7	29.0
	Kimono	44.2	80.5	-	69.3	49.5	38.1
	ParkScene	43.0	40.0	33.2	39.7	47.4	31.6
1280 × 720 Class E	FourPeople	51.0	51.7	32.2	42.3	48.9	29.8
	Johnny	53.6	67.9	-	57.2	49.9	46.7
	KristenAndSara	62.7	63.5	-	55.5	49.5	43.7
832 × 480 Class C	BasketballDrill	41.0	39.7	-	48.2	48.7	31.0
	BQMall	44.0	38.3	-	43.3	47.0	-
	PartyScene	40.7	28.8	33.4	49.4	41.1	-
	RaceHorses	44.1	-	32.4	46.1	44.6	31.0
416 × 240 Class D	BasketballPass	40.5	45.9	29.4	47.0	46.8	31.0
	BlowingBubbles	40.7	36.2	32.2	41.3	44.2	-
	BQSquare	41.0	27.9	-	47.1	41.0	14.5
	RaceHorses	41.9	-	-	-	46.5	-
All class	**Average**	45.33	48.02	31.9	47.0	47.0	32.6

**Table 7:** The BDBR of the proposed method compared with the anchor method and previous works.

Class	Test Sequence	Proposed	[22]	[24]	[25]	[34]	[35]
		BD-Rate (%)	BD-Rate (%)	BD-Rate (%)	BD-Rate (%)	BD-Rate (%)	BD-Rate (%)
2560 × 1600 Class A	Traffic	0.91	0.98 *	0.54	-	1.46	-
	PeopleOnStreet	1.15	1.20	-	-	1.71	-
1920 × 1080 Class B	BasketballDrive	0.87	1.87	1.21	2.3	2.37	0.89
	BQTerrace	0.67	1.05	-	2.6	0.82	0.83
	Cactus	1.00	1.02	-	2.9	1.46	0.91
	Kimono	1.55	3.72	-	0.8	1.54	0.75
	ParkScene	0.94	0.67 *	0.87	0.5	1.02	1.07
1280 × 720 Class E	FourPeople	1.17	1.70 *	1.45	2.7	1.78	1.23
	Johnny	1.20	3.01	-	1.5	2.22	0.96
	KristenAndSara	1.55	2.39	-	1.1	2.21	0.79
832 × 480 Class C	BasketballDrill	0.72	0.99 *	-	0.8	0.85	0.90
	BQMall	1.10	1.07	-	2.4	1.47	-
	PartyScene	1.17	0.24	1.23	2.0	1.02	-
	RaceHorses	0.70	-	0.94	1.0	0.65	0.91
416 × 240 Class D	BasketballPass	1.11	1.34	0.26	0.3	1.71	0.87
	BlowingBubbles	1.11	0.50 *	0.21	0.4	1.03	-
	BQSquare	1.40	0.48	-	1.0	1.29	0.34
	RaceHorses	0.94	-	-	-	1.22	-
All class	**Average**	1.07	1.39	0.83	1.5	1.44	0.87

**Table 8:** Encoding performance of the proposed method compared with the anchor method and previous works.

Class	Test Sequence	Proposed	[22]	[24]	[25]	[34]	[35]
**All class**	**BDBR**	1.07	1.39	0.83	1.50	1.44	0.87
	**TS**	45.33	48.02	31.90	47.00	47.00	32.60
	**TS/BDBR**	42.36	34.54	38.43	31.33	32.63	37.47

**Table 9:** Encoding performance of the proposed overall method compared with the anchor method and previous works for six visual sensor video sequences.

Video	Proposed		[22]		[34]	
	BD-Rate (%)	TS (%)	BD-Rate (%)	TS (%)	BD-Rate (%)	TS (%)
FourPeople	1.17	51.0	1.70 *	51.8	1.78	48.9
Johnny	1.20	53.6	3.01	68.0	2.22	49.9
KristenAndSara	1.55	62.7	2.39	63.6	2.21	49.5
Vidyo1	1.04	51.0	2.54	62.0	1.98	49.6
Vidyo3	0.77	51.6	3.15	64.3	1.49	50.2
Vidyo4	1.05	52.4	1.89	59.2	1.74	48.1
**Average**	1.13	53.72	2.44	61.48	1.90	49.37

Note: The symbol * indicates that some frames of a sequence were used in the training set in [22].

## Data Availability

Not applicable.
